# LegNER: a domain-adapted transformer for legal named entity recognition and text anonymization

**DOI:** 10.3389/frai.2025.1638971

**Published:** 2025-11-06

**Authors:** Ioannis Karamitsos, Nikolaos Roufas, Khalil Al-Hussaeni, Andreas Kanavos

**Affiliations:** 1 Department of Graduate Programs and Research, Rochester Institute of Technology, Dubai, United Arab Emirates; 2 Department of Informatics, Ionian University, Corfu, Greece; 3 Department of Computing Sciences, Rochester Institute of Technology, Dubai, United Arab Emirates

**Keywords:** legal NLP, named entity recognition, transformer models, text anonymization, domain adaptation, GDPR compliance

## Abstract

The increasing demand for scalable and privacy-preserving processing of legal documents has intensified the need for accurate Named Entity Recognition (NER) systems tailored to the legal domain. In this work, we introduce **LegNER**, a domain-adapted transformer model designed for both legal NER and text anonymization. The model is trained on a corpus of 1,542 manually annotated court cases and enriched with an extended legal vocabulary, enabling robust recognition of six critical entity types, including PERSON, ORGANIZATION, LAW, and CASE_REFERENCE. Built on BERT-base and enhanced through domain-specific pretraining and span-level supervision, LegNER consistently outperforms established legal NER baselines. Experimental results demonstrate significant gains in accuracy (99%), F1 score (over 99%), and inference efficiency (processing more than 12 documents per second), confirming both its precision and scalability. Beyond quantitative improvements, qualitative evaluation highlights LegNERs ability to generate coherent anonymized outputs, a crucial requirement for GDPR-compliant redaction and automated legal analytics. Taken together, these results establish LegNER as a reliable and effective solution for high-precision entity recognition and anonymization in compliance-sensitive legal workflows.

## 1 Introduction

The digital transformation of justice systems is reshaping how legal services are delivered, creating opportunities to enhance transparency, accessibility, and operational efficiency across courts and legal institutions (Demertzis et al., 2023; Ruggeri et al., 2022; Sinyakin et al., 2020). This transformation is part of a broader shift toward digitized governance, where technologies such as natural language processing (NLP), blockchain, and explainable artificial intelligence (XAI) support legal decision-making, foster public trust, and enable accountable automation (Bhattacharya et al., 2019; Demertzis et al., 2023; Oussalah, 2021; Sharma et al., 2018). Recent progress in conversational agents and legal document summarization further underscores the demand for robust semantic processing of legal texts (Amato et al., 2023; Andrade and Becker, 2023; Mouratidis et al., 2022). Within this landscape, Named Entity Recognition (NER) plays a foundational role by enabling downstream applications such as summarization, classification, and anonymization, including comparative analyses of extractive and abstractive summarization workflows (Giarelis et al., 2023).

Beyond these application-driven motivations, it is essential to situate our work within the broader international research landscape. Named Entity Recognition (NER) and text anonymization have been extensively studied across legal and administrative domains in multiple languages. Foundational English-language resources include large-scale court decision corpora and legal-judgment anonymization studies (Chalkidis et al., 2020; Ferraro et al., 2019). At the same time, significant progress has been reported in other languages and multilingual contexts, such as Portuguese (Avram et al., 2021), Hindi (Kapoor et al., 2022), and cross-lingual European corpora like PyEuroVoc and EuroCases (Giampieri, 2023; Wu et al., 2023). These efforts illustrate both the maturity of NER and anonymization methods and the persistent challenges of adapting them to diverse legal systems.

Beyond summarization, machine learning models have been applied to tasks such as classifying legal articles by sector, reflecting the intersection of entity recognition and thematic categorization in regulatory contexts (Yang et al., 2023). Studies on multilingual COVID-19 legislation illustrate both the potential and limitations of automated approaches in high-stakes regulatory environments (Egger et al., 2024), while advances in legal citation classification confirm the feasibility of domain-specific neural models for structured legal text analysis (Xie et al., 2024).

Despite these advances, the exponential growth of legal texts presents persistent challenges. Legal documents are characterized by complex syntax, domain-specific jargon, multilingual variation, and jurisdiction-dependent semantics (Moreno-Schneider et al., 2020; Rodŕıguez-Puente and Hernández-Coalla, 2023). Such properties limit the effectiveness of traditional text analysis methods, especially for downstream tasks such as citation extraction, summarization, and anonymization. Legal language is simultaneously rigid and ambiguous, with redundant phrasing and intertextual references that complicate computational processing. Translation inconsistencies further hinder information extraction across multilingual legal systems (Giampieri, 2023; Wu et al., 2023).

A critical application of legal NLP is the anonymization of court decisions–essential not only for preserving privacy and complying with regulations such as the GDPR, but also for enabling open justice by releasing accessible judgments without disclosing sensitive information (Dorfleitner et al., 2023; Savelka and Ashley, 2023). NER is a key enabler of this process, as it allows for the identification of litigants, judges, institutions, and statutory references in unstructured text (Kapoor et al., 2022; Nguyen et al., 2022; Sang and De Meulder, 2003). When integrated into anonymization pipelines, legal NER supports the secure publication of legal texts while safeguarding privacy and transparency.

However, general-purpose NER systems struggle with nuanced legal expressions, multi-word entities, and rare domain terms that are critical in legal texts. To address this gap, we propose **LegNER**, a domain-adapted transformer model for legal named entity recognition and text anonymization. LegNER builds on BERT-base, incorporates legal-domain pretraining, and is fine-tuned on curated legal corpora including statutes, contracts, and court decisions (Alves et al., 2023; Avram et al., 2021; Paul et al., 2022). The model is integrated into an anonymization pipeline capable of masking sensitive information in court rulings, enabling privacy-preserving automation in legal workflows.

Legal NER enhances not only anonymization but also semantic search, legal citation extraction, e-discovery, and decision prediction, supporting lawyers, scholars, and regulators in navigating large volumes of unstructured legal text (Alcántara Francia et al., 2022; Ferraro et al., 2019; Paul et al., 2022; Rossi and Palmirani, 2015). These applications underscore the importance of domain-adapted NER models as building blocks for scalable, interpretable legal AI.

Nonetheless, domain-specific NER faces major challenges: limited annotated training data, the need for multilingual and jurisdiction-specific adaptation, and risks of bias in legal AI systems (Al-Qurishi et al., 2022; Hua et al., 2023; Skianis et al., 2020; Song et al., 2022; Tsarapatsanis and Aletras, 2021; Wu et al., 2023). Ethical concerns such as algorithmic opacity, fairness in automated annotations, and responsible data use must also be addressed (Eiermann, 2024; Wachter et al., 2017). Transparency in model design, explainability of outputs, and risk-aware deployment are therefore essential for trustworthy integration into legal infrastructures.

Recent surveys highlight persistent limitations in automating legal reasoning, knowledge representation, and judicial decision support (Zhong et al., 2020). Against this backdrop, modular and interpretable components like LegNER represent concrete steps toward more comprehensive and responsible legal AI.

Despite significant progress, existing legal NER and anonymization systems leave important needs unmet. Models such as Legal-BERT (Chalkidis et al., 2020), LegalNER.pt (Avram et al., 2021), and LEDGAR-based approaches (Tuggener et al., 2020) demonstrate strong performance in named entity extraction, but they generally lack integrated mechanisms for fine-grained span alignment, automated anonymization evaluation, and cross-jurisdiction adaptation. LegNER directly addresses these shortcomings by combining domain-specific pretraining, span-level supervision, and dedicated anonymization metrics in a single, reproducible pipeline. This integrated design fills a critical gap between high-performing NER systems and real-world privacy-compliant legal workflows.

This paper makes the following key contributions to the field of legal NLP:

1. We introduce **LegNER**, a domain-adapted transformer model for legal NER, and implement four transfer learning strategies within an end-to-end pipeline for entity classification and anonymization in court documents.
2. We provide a comprehensive evaluation across multiple benchmark legal corpora, demonstrating state-of-the-art performance in accuracy, precision, recall, and F1-score, and analyzing training convergence, entity-level behavior, and anonymization consistency.
3. We validate LegNER's applicability in real-world legal workflows through qualitative assessments and anonymization case studies, confirming its robustness for GDPR-compliant processing and privacy-aware deployment in judicial and regulatory contexts.

The remainder of this paper is organized as follows: Section 2 reviews prior work on legal NER and domain-adapted transformers. Section 3 introduces the legal annotation schemes used in our study. Section 4 describes the design of the LegNER system and its anonymization pipeline. Section 5 details the corpus construction, transfer learning strategies, and implementation setup. Section 6 presents experimental results, including comparative benchmarking, training analysis, and qualitative evaluation. Finally, Section 8 summarizes findings and outlines directions for future research in legal AI and privacy-preserving document processing.

## 2 Related work

Legal NER and text anonymization have developed into active areas of international research over the past decade. In English, widely used legal corpora and benchmark tasks–ranging from U.S. case law to European legislative documents–have provided fertile ground for NER innovation (Chalkidis et al., 2020; Ferraro et al., 2019). Parallel efforts in other languages, including Portuguese (Avram et al., 2021), Hindi (Kapoor et al., 2022), and multilingual European resources such as EuroCases and PyEuroVoc (Giampieri, 2023; Wu et al., 2023), confirm that the problem is not confined to any single jurisdiction. These multilingual studies demonstrate the benefits of cross-lingual transfer while highlighting the difficulty of maintaining consistent annotation standards and entity definitions across legal systems.

The rapid digitalization of legal systems has significantly increased the volume, complexity, and heterogeneity of legal documents. This has created a pressing need for automated tools capable of extracting actionable information from legal texts. Within this context, Natural Language Processing (NLP) and, in particular, Named Entity Recognition (NER) have emerged as foundational techniques. NER enables the detection and classification of entities such as litigants, judges, institutions, statutes, and case references (Kapoor et al., 2022; Nguyen et al., 2022; Sang and De Meulder, 2003). Yet the domain-specific characteristics of legal language–formality, jurisdictional variation, long compound expressions, and frequent citations–pose challenges that generic NLP systems often fail to address adequately (Kanavos et al., 2023a; Mohasseb et al., 2025; Vonitsanos et al., 2023).

Early legal NER systems were rule-based or pattern-matching approaches, typically developed for narrow tasks such as case indexing or citation retrieval. While effective in limited contexts, these methods lacked scalability and adaptability. The introduction of machine learning, particularly Conditional Random Fields (CRFs) and later neural models, improved flexibility and accuracy (Alves et al., 2023; Avram et al., 2021). A major breakthrough came with transformer architectures such as BERT and RoBERTa, which significantly advanced performance. However, general-purpose pretrained models still suffer from vocabulary mismatch, semantic drift, and domain misalignment in legal settings (Chalkidis et al., 2020; Hua et al., 2023; Paul, 2023).

To overcome these limitations, domain-adapted models have been proposed. Legal-BERT, pretrained on legal corpora, improved semantic coverage and downstream task performance (Zhong et al., 2020). Similarly, LegalNER.pt, trained on Portuguese legal texts, illustrates the benefits of jurisdiction-specific adaptation. These efforts highlight the importance of transfer learning and domain-aware training (Al-Qurishi et al., 2022; Sang and De Meulder, 2003). Despite progress, cross-jurisdictional transfer remains difficult due to syntactic differences, varied institutional references, and specialized terminology. Comparable challenges have been observed in cross-domain retrieval tasks, where legal and patent texts diverge structurally and lexically (Althammer et al., 2021).

The availability of annotated corpora has been pivotal for progress. Datasets such as CoCo-LEG for contract classification, HLDC for Hindi legal texts, and CoCELD for comparative legal language change provide important evaluation benchmarks (Avram et al., 2021; Kapoor et al., 2022; Rodŕıguez-Puente and Hernández-Coalla, 2023; Stow et al., 2023). European initiatives like EuroCases and the ECLI network have promoted standardized open resources, while PyEuroVoc and multilingual datasets have enabled cross-lingual research (Giampieri, 2023; Wu et al., 2023). Nevertheless, many legal systems–particularly those in under-resourced languages–lack publicly available annotated corpora, limiting comparative studies and model generalization.

Beyond these representative examples, a rich landscape of legal NER datasets now supports multilingual research. Well-established English corpora include the Caselaw Access Project and the LEDGAR contract dataset (Tuggener et al., 2020), while German resources such as GerDaLIR and the German Federal Court corpus provide jurisdiction-specific annotations. Spanish and Portuguese are covered by IberLegal and LegalNER.pt (Avram et al., 2021), Italian by Ita-CaseLaw, and Romanian by RONEC-Legal. Comparative resources like CoCo-LEG, HLDC, and CoCELD (Avram et al., 2021; Kapoor et al., 2022; Rodŕıguez-Puente and Hernández-Coalla, 2023; Stow et al., 2023) further extend cross-lingual coverage. Together these corpora demonstrate substantial progress in data availability, even as heterogeneity in annotation guidelines and gaps in certain legal subdomains continue to challenge full interoperability.

Legal NER has been integrated into practical applications including document anonymization, compliance monitoring, semantic search, and litigation analytics. For instance, ensemble learning methods have been applied to legal document similarity tasks, leveraging entity-level features for improved retrieval and case linkage (Fan et al., 2024). Anonymization of judicial decisions remains a critical application for GDPR-compliant public access (Dorfleitner et al., 2023; Savelka and Ashley, 2023), with NER serving as the backbone of redaction pipelines. Studies in GDPR compliance, legal question answering, and document classification confirm the central role of entity extraction in both privacy protection and usability (Ferraro et al., 2019; Paul et al., 2022).

At the architectural level, enhancements such as hierarchical token alignment, span-based modeling, and contextualized embeddings tuned for citation patterns have been explored to improve legal NER (Huang et al., 2022; Skianis et al., 2020; Sugathadasa et al., 2019). Hybrid systems that combine symbolic rules with neural architectures have gained attention for their interpretability, though scalability remains an obstacle. Complementary approaches based on algebraic or logic-based methods have also been investigated for tasks such as legal rule modeling and structural verification (Letychevskyi et al., 2022). In parallel, OCR preprocessing challenges persist in digitizing historical archives, where poor scan quality can significantly degrade NER performance (Savelka and Ashley, 2023).

Large generative models such as GPT-3 and instruction-tuned LLMs have recently been applied to legal NLP tasks, including clause drafting and contract generation (Lin and Cheng, 2024). While they offer impressive zero-shot capabilities, studies reveal that they struggle with fine-grained legal entity recognition unless supported by tailored prompts and postprocessing (Eiermann, 2024; Wachter et al., 2017). Effective adaptation still requires finetuning, vocabulary reconfiguration, or carefully engineered prompting strategies.

Finally, ethical concerns around fairness, transparency, and accountability in legal NLP have become increasingly prominent. Biases in training data, the opacity of neural models, and the high stakes of legal decision-making amplify risks (Eiermann, 2024; Tsarapatsanis and Aletras, 2021). To ensure trustworthiness, legal AI systems must adhere to principles of due process and interpretability, allowing automated annotations to be audited and contested. Regulatory frameworks and professional standards are therefore essential for guiding responsible deployment.

In summary, existing research has established the importance of domain adaptation, dataset quality, and architectural innovation for legal NER. Yet challenges persist in multilingual adaptation, real-world anonymization, and ethical deployment. In this study, we present **LegNER**, a domain-adapted transformer model trained on curated legal corpora and evaluated across four transfer learning strategies. Our work addresses entity granularity, jurisdictional specificity, and anonymization robustness, contributing to the development of reliable, transparent, and ethically aligned legal NLP systems.

Building on these observations, while prior legal-domain transformers and multilingual NER systems (Zhong et al., 2020; Chalkidis et al., 2020; Avram et al., 2021; Kapoor et al., 2022; Tuggener et al., 2020) have advanced entity recognition, they provide limited support for end-to-end anonymization and often lack mechanisms for iterative feedback and span-level consistency. LegNER is designed to bridge this gap by unifying legal-domain pretraining, fine-grained entity modeling, and GDPR-oriented anonymization assessment within a single framework.

## 3 Preliminaries

As legal systems worldwide undergo digital transformation, they generate increasingly large volumes of complex, unstructured textual data. Extracting structured information from these texts is essential for applications in legal analytics, regulatory compliance, and document anonymization. This section outlines the foundations of Legal Natural Language Processing (Legal NLP), focusing on Named Entity Recognition (NER) and transformer-based models, while also discussing the unique linguistic and technical challenges of processing legal documents at scale.

### 3.1 Overview of legal NLP and NER

The rapid digitalization of judicial systems has introduced a surge of complex legal texts that demand automated understanding and semantic interpretation. Within this landscape, Natural Language Processing (NLP) techniques—and particularly Named Entity Recognition (NER)—have become fundamental tools for structuring unstructured legal data. NER refers to the task of identifying and classifying textual spans into predefined categories such as PERSON, ORGANIZATION, LOCATION, and LAW entities (Sang and De Meulder, 2003). In the legal domain, these categories are further extended to case references, court names, statutory citations, and procedural acts (Al-Qurishi et al., 2022; Chalkidis et al., 2017; Kapoor et al., 2022).

NER supports downstream tasks such as anonymization, legal search, citation graph construction, and contract analytics. For instance, anonymization systems relying on NER can systematically remove or mask sensitive personal identifiers in public court decisions, thus facilitating compliance with data protection frameworks like the GDPR (Dorfleitner et al., 2023; Ruggeri et al., 2022; Tsarapatsanis and Aletras, 2021). However, general-purpose NLP pipelines are insufficient for such specialized use cases. Legal texts are characterized by archaic constructions, jurisdiction-specific expressions, and nested references that add high syntactic and semantic complexity (Rodŕıguez-Puente and Hernández-Coalla, 2023). This makes domain-adapted NER pipelines, trained on representative legal corpora, essential for scalable and accurate automation (Avram et al., 2021; Wachter et al., 2017).

### 3.2 Challenges in legal document processing

Legal NER faces unique challenges related to domain complexity and variability. First, the syntactic structure of legal documents is highly non-canonical. Sentences are typically long, clause-heavy, and interleaved with references, complicating sentence boundary detection and token-level annotation (Chalkidis et al., 2020; Leitner et al., 2019). Even human readers—particularly non-native speakers—struggle with these features, underscoring the linguistic opacity of legal texts (Al-Jarf, 2023).

Second, terminology varies significantly across jurisdictions and document types. Terms such as “plaintiff,” “claimant,” or “applicant” may be used interchangeably in different systems, complicating generalization. Entities are also frequently expressed in abbreviated, partial, or referential forms. Coreference resolution, the task of linking such mentions to the same entity, remains a major limitation of current systems (Chalkidis et al., 2017; Chang et al., 2021; Eiermann, 2024).

Third, while substantial progress has been made in creating annotated corpora for legal NER, important gaps remain. Well-known resources include HLDC (Kapoor et al., 2022), CoCELD (Rodŕıguez-Puente and Hernández-Coalla, 2023), and LEDGAR (Tuggener et al., 2020) for English, as well as multilingual and jurisdiction-specific datasets such as LegalNER.pt for Portuguese (Avram et al., 2021), GerDaLIR for German, IberLegal for Spanish, Ita-CaseLaw for Italian, and RONEC-Legal for Romanian. These corpora enable large-scale training and benchmarking, but their coverage and annotation conventions differ widely, and certain languages and specialized subdomains are still underrepresented. This unevenness continues to challenge cross-lingual model transfer and hinders fully universal legal NER.

Finally, digitization of historical archives often introduces OCR-related noise, which further reduces NER accuracy by distorting entity boundaries (Savelka and Ashley, 2023; Skianis et al., 2020).

### 3.3 Role of transformer models in legal NLP

Transformer-based models have fundamentally reshaped NLP by offering contextualized representations through self-attention and bidirectional encoding. BERT (Bidirectional Encoder Representations from Transformers), in particular, has set new performance standards across NLP tasks, including legal NER (Chalkidis et al., 2020; Li and Zhang, 2021). Unlike RNN-based systems, transformers process sequences in parallel while modeling long-range dependencies, which is essential for lengthy legal documents containing distant coreferent mentions (Garg et al., 2023; Roufas et al., 2025).

In the legal domain, pretrained transformer variants such as Legal-BERT, CaseLawBERT, and ContractBERT have been developed to overcome the shortcomings of generic models. Trained on large-scale legal corpora, these models exhibit improved performance in tasks such as entity classification, statute referencing, and document summarization (Hua et al., 2023; Paul et al., 2022; Zhong et al., 2020).

**Figure 1:**
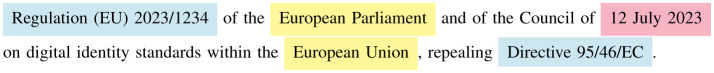
Example of NER tagging in a legal sentence. Detected entities include LAW (Regulation (EU) 2023/1234 and Directive 95/46/EC), ORG (European Parliament and European Union), and DATE (12 July 2023).

Figure 1 illustrates the output of a domain-specific NER model applied to a legal sentence. The model successfully identifies entities including a regulation title (LAW), an institutional actor (ORG), and a reference date (DATE), demonstrating how structured knowledge can be extracted from raw legal text.

Despite their strengths, transformer models face practical constraints. They demand large computational resources and substantial amounts of labeled data, limiting their applicability in resource-constrained or multilingual environments. Moreover, zero-shot performance on specialized legal texts remains poor unless models are fine-tuned on in-domain data (Huang et al., 2022; Song et al., 2022).

To address these limitations, our study implements domain-adapted transformers using multi-phase transfer learning. By leveraging legal-specific annotation schemes, span-aware token alignment, and vocabulary curation, LegNER bridges the gap between general-purpose NLP models and the linguistic complexities of legal text. The architecture and methodology are detailed in the following sections.

## 4 LegNER architecture and functionality

The **LegNER** system is a domain-adapted Named Entity Recognition (NER) framework tailored for legal document processing. It addresses challenges such as jurisdiction-specific terminology, long cross-referential constructs, and anonymization constraints through a modular pipeline of four phases: corpus construction, pretraining, fine-tuning, and evaluation. These stages are reusable and adaptive, enabling incremental improvements as annotated data or error feedback become available.

**Figure 2:**
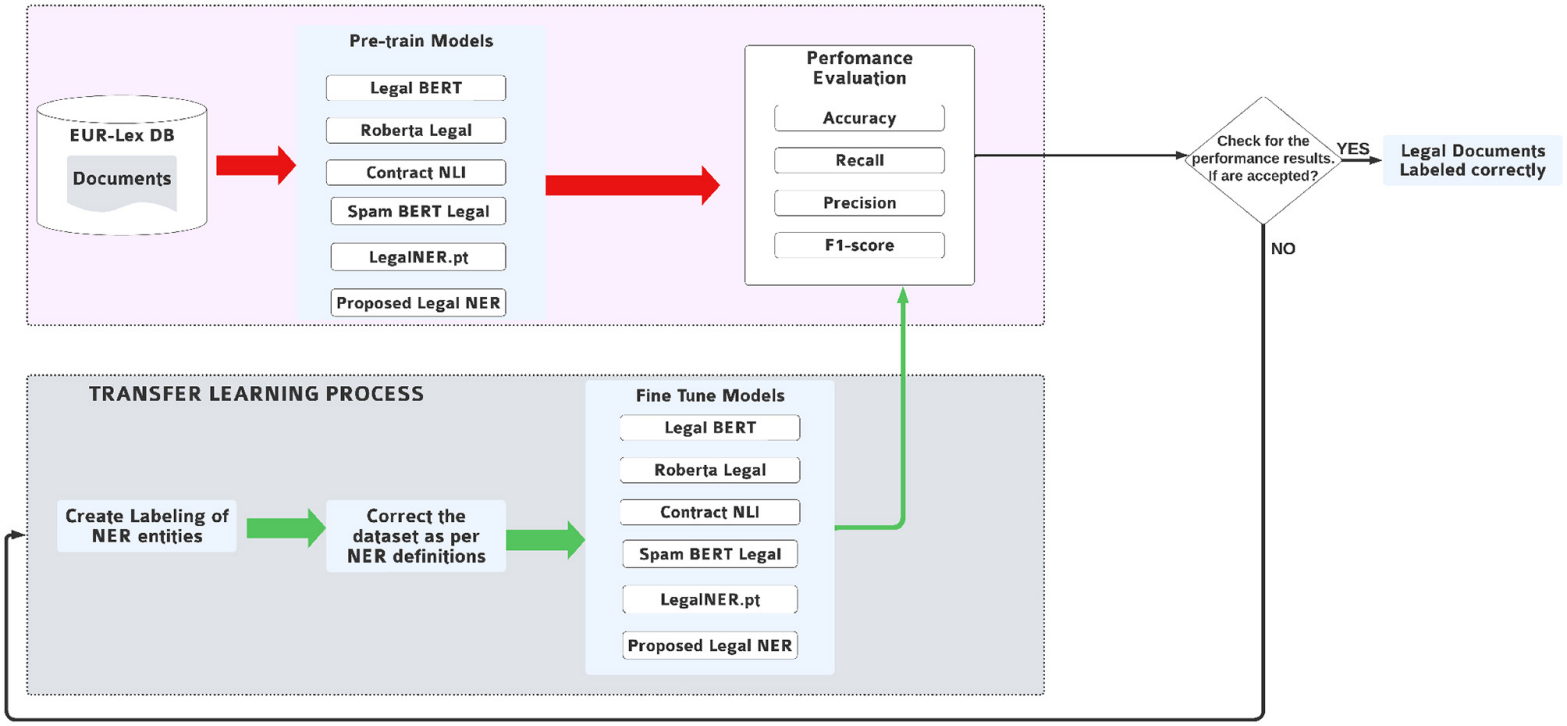
LegNER architecture with unified baseline model names: Legal-BERT, RoBERTa-Legal, ContractNLI, SpanBERT-Legal, and LegalNER.pt.

Figure 2 illustrates the overall architecture. The upper part of the diagram shows the sequential flow of data from raw legal documents to performance assessment, while the lower part highlights a feedback loop that supports iterative learning and parameter refinement.

Each phase plays a distinct role in enhancing the accuracy and generalizability of the system:

- **Corpus Construction:** Legal documents are collected from authoritative sources such as EUR-Lex, cleaned and segmented, and filtered to remove OCR noise and formatting artifacts. Annotated entities relevant to anonymization include PERSON, LAW, CASE_REFERENCE, ORG, and LOCATION. Annotations are stored in span-based JSON structures compatible with standard tokenizers.
- **Pretraining:** The selected corpus is used to pretrain a transformer-based language model via masked language modeling (MLM). Models such as Legal-BERT, RoBERTa-Legal, and SpanBERT-Legal are initialized and trained on unannotated legal text to encode domain-specific syntax and semantics (Chalkidis et al., 2020; Hua et al., 2023). The demonstrated ability of large-scale models such as GPT-4 to solve complex legal reasoning tasks further illustrates the value of transformer pretraining in law (Katz et al., 2024).
- **Fine-Tuning:** The pretrained model is fine-tuned on the annotated corpus with a token classification head and optimized using cross-entropy loss over BIO-tagged spans. Alignment ensures entity boundaries are preserved during subword tokenization. Hyperparameters such as learning rate, batch size, and number of epochs are tuned on validation sets for balanced precision and recall.
- **Evaluation and feedback:** Performance is measured using precision, recall, and F1, complemented by domain-specific criteria such as consistency (uniform tagging of repeated mentions) and readability (semantic coherence post-anonymization). Errors identified–such as boundary mismatches or missed abbreviations–are integrated back into the annotation and training pipeline for incremental refinement.

This modular design allows LegNER to scale across jurisdictions and subdomains. For example, if new entities are required (e.g., JUDGE_NAME, ARTICLE_REF), only the annotation schema and classification head need modification. The remainder of the pipeline remains reusable, ensuring efficient adaptation to multilingual or evolving legal contexts.

## 5 Methodology and implementation details

This section details the methodological framework underlying the development and evaluation of LegNER. The process is divided into five phases: dataset construction, transformer pretraining, supervised fine-tuning, baseline model setup, and quantitative evaluation. Each phase is informed by best practices in domain-specific transfer learning and legal NLP engineering.

### 5.1 Dataset construction and annotation

The EUR-Lex platform^1^ provides access to a comprehensive repository of European Union legal documents, including treaties, regulations, directives, case law, and legal opinions. It was chosen for this study because of its multilingual coverage, legal diversity, and consistent structure, which have also been leveraged in prior research on legal discourse and parallel corpora addressing sensitive topics such as hate speech and incitement (Giordano et al., 2023).

Although EUR-Lex is a multilingual database covering all official EU languages, the present study uses the English versions of legal documents for annotation and training. The term *multilingual EU dataset* refers to the corpuss potential cross-lingual applicability, since the same documents exist in multiple languages and could support future multilingual or cross-lingual adaptation.

To build our dataset we first queried EUR-Lex for published court decisions between 2015 and 2023 using a combination of keywords related to data protection, administrative law, and competition policy. Only documents with stable identifiers, complete rulings, and machine-readable XML were retained. After de-duplication and removal of purely procedural notes, 1,542 full-text judgments were selected.

This subset was curated specifically for the task of legal NER and anonymization. The cases span domains such as data protection, administrative law, and competition policy, ensuring a representative distribution of entity types and structural patterns. Legal documents introduce unique linguistic challenges–including jurisdiction-specific terminology, archaisms, and long syntactic constructs–that must be considered during annotation and model adaptation (Beebeejaun, 2023).

Each document was manually or semi-automatically annotated for six key entity categories:

- PERSON—names of individuals involved in proceedings (e.g., judges, plaintiffs, lawyers)
- ORGANIZATION—institutions such as commissions, corporations, and NGOs
- LOCATION—cities, countries, and jurisdictions
- DATE—calendar dates in multiple formats
- LAW—statutes, directives, regulations, and legal articles
- CASE_REFERENCE—case identifiers and procedural citations

Annotation proceeded in two stages. About 600 documents (39%) were fully annotated by trained legal experts. The remaining 942 documents underwent semi-automatic pre-labeling based on rule-based lexicons, regular-expression patterns, and weak NER baselines, after which all suggestions were manually verified and corrected. This hybrid procedure accelerated annotation while preserving expert-level accuracy.

The dataset was divided into training (70%, 1,079 documents), validation (15%, 231 documents), and test (15%, 232 documents) sets using stratified sampling. This ensured proportional representation of all categories (e.g., PERSON, LAW, CASE_REFERENCE) across subsets, preventing bias from overrepresented entities and reducing the risk of data leakage. The training set supported model fine-tuning, the validation set was used for early stopping and hyperparameter tuning, and the test set was reserved strictly for final evaluation.

Overall, the corpus contains roughly 34,000 sentences and 1.2 million tokens, with 287,000 annotated entity mentions. Table 1 reports the frequency of each entity type, confirming balanced coverage across all categories.

**Table 1:** Entity statistics for the LegNER Dataset.

**Entity type**	**Number of mentions**
PERSON	72,400
ORGANIZATION	51,800
LOCATION	42,300
DATE	64,900
LAW	33,500
CASE_REFERENCE	22,100

Annotations follow a span-based format, capturing not only the entity text but also its exact character-level offsets in the source document. This is essential for alignment with subword tokenizers used in models such as BERT and RoBERTa, where token boundaries rarely match word boundaries (Kapoor et al., 2022; Rodŕıguez-Puente and Hernández-Coalla, 2023). Each annotation records the entity type, start and end indices, and entity span. This structured approach enables precise training and evaluation by supporting exact and overlapping entity matches. Prior work in extracting structured information from legal contracts–including obligations, parties, and temporal markers–has shown the importance of span-level representations for capturing semantic granularity in legal texts (Chalkidis et al., 2017).

The following listing shows a typical annotated legal sentence in JSON format, where a named entity recognition system must identify legal actors, institutional bodies, law references, and temporal or geographic markers:

**JSON Annotation for Legal NER.**

{ document_id: case_001, text: On March 15, 2023, Judge Alice Thompson ruled in favor of the European Commission in case C-123/20 concerning GDPR compliance in Berlin., entities: [ type: DATE, start: 3, end: 18, text: March 15, 2023, type: PERSON, start: 26, end: 42, text: Alice Thompson, type: ORGANIZATION, start: 61, end: 82, text: European Commission, type: CASE_REFERENCE, start: 86, end: 95, text: C-123/20, type: LAW, start: 109, end: 114, text: GDPR, type: LOCATION, start: 130, end: 136, text: Berlin ] }

Each entity type plays a distinct role in legal workflows. For instance, LAW links to legislative databases, PERSON and ORGANIZATION are central to anonymization, and CASE_REFERENCE supports precedent retrieval. Accurate span-level labeling thus enables systematic extraction for downstream tasks such as semantic search, citation graph construction, and GDPR-compliant redaction.

### 5.2 Transfer learning pipeline

The LegNER system implements a structured transfer learning pipeline that operationalizes the architecture described in Section 4. The pipeline is divided into two interconnected phases: a static data flow layer, responsible for preparing model-ready inputs, and a dynamic feedback loop, which introduces adaptability and iterative refinement. This design ensures that LegNER can simultaneously leverage large volumes of unlabeled legal data and systematically address error patterns emerging during supervised training.

**Phase 1 (upper layer: static data flow)**. This stage encompasses corpus ingestion, text preprocessing, annotation, and domain-adapted pretraining (Dritsas et al., 2019; Kanavos et al., 2023b). It represents a one-way flow from raw documents to pretrained representations, where key steps include:

- **Text normalization:** Removal of OCR noise, repeated headers and footers, and redundant metadata, which are common in legal corpora digitized from heterogeneous sources.
- **Annotation:** Manual and semi-automatic span-level labeling, supported by legal dictionaries and signal phrase patterns (van der Veen and Sidorova, 2021), augmented by semi-supervised models to bootstrap low-frequency entity classes.
- **Domain-specific pretraining:** Large-scale Masked Language Modeling (MLM) on unannotated EUR-Lex documents to capture co-occurrence patterns of statutes, case citations, and institutional entities. This stage is critical for reducing vocabulary mismatch and semantic drift observed in general-purpose models when applied to legal corpora.

**Phase 2 (Lower layer: dynamic learning loop)**. While the static layer establishes strong initial representations, domain-specific adaptation requires iterative refinement. The lower layer introduces supervised fine-tuning and error-driven feedback to close this gap:

- **Fine-tuning:** Supervised training on the annotated dataset using token-level classification, aligning entity spans with subword token boundaries. This stage adapts pretrained embeddings to the specific granularity of legal NER tasks.
- **Performance assessment:** Standard evaluation metrics (precision, recall, F1) are complemented by domain-specific criteria such as consistency across repeated mentions and readability after anonymization (see Section 6).
- **Feedback integration:** Common failure cases–including nested entities (e.g., “Court of Justice of the European Union” within a longer reference), ambiguous abbreviations, and incorrect span boundaries–are logged and reintegrated into the annotation guidelines or training corpus. This error-aware loop enables LegNER to improve incrementally without the need for exhaustive reannotation.

The separation of static and dynamic layers reflects a balance between stability and adaptability. The static flow leverages the breadth of unannotated legal texts to provide robust initial embeddings, while the dynamic loop introduces resilience by correcting systematic errors in supervised training. Together, these phases create a flexible transfer learning framework capable of scaling across legal domains and jurisdictions.

### 5.3 Baseline configurations for comparative evaluation

To assess the effectiveness of LegNER, we benchmarked its performance against a diverse set of state-of-the-art NER architectures widely applied in both general-domain and legal-domain NLP. These baselines provide direct points of comparison with established transformer variants and help isolate the incremental benefit of domain adaptation for legal text processing.

The selected baselines include **Legal-BERT**^2^ (Chalkidis et al., 2020), **RoBERTa-Legal**^3^, **SpanBERT-Legal**^4^, **ContractNLI**^5^ (Koreeda and Manning, 2021), and **LegalNER.pt**^6^ (Avram et al., 2021), which is a BERT-based model pretrained on large Portuguese legal corpora and fine-tuned on our EU-law dataset to assess cross-lingual robustness. For comparison, we also include general-domain transformer variants such as **BERT-base** (cased and uncased) and **T5** (Raffel et al., 2020). All models were implemented using the Hugging Face Transformers library (Wolf et al., 2020), ensuring a consistent training interface and reproducible fine-tuning procedures. The set reflects diversity along three key dimensions:

- **Tokenizer and vocabulary design:** WordPiece (BERT variants), byte-pair encoding (RoBERTa), and SentencePiece (T5) differ in how they segment rare or domain-specific terms, directly influencing span boundary accuracy in legal NER.
- **Pretraining corpus:** Some baselines are pretrained on generic corpora (BERT-base), while others incorporate legal-domain text such as Legal-BERT, RoBERTa-Legal, and LegalNER.pt. This allows us to evaluate the effect of domain relevance in pretraining.
- **Architectural emphasis:** SpanBERT-Legal focuses on span-level embeddings, T5 follows a sequence-to-sequence paradigm suited for generative tasks, and ContractNLI represents a contract-specific transformer for fine-grained legal relations.

Comparing across these paradigms highlights the trade-offs between generative and discriminative NER approaches as shown in Table 2.

**Table 2:** Architectural and training configurations of baseline legal NER models.

**Base architecture**	**Tokenizer and vocabulary**	**Max sequence length**	**Batch size**	**Learning rate**	**Optimizer**	**Special features**
BERT-base	30,522 tokens + 1,245 legal terms	512	32	3e-5	AdamW	Legal terms optimization
BERT-base-uncased	50,000+ tokens	512	32	2e-5	Adam	Pretrained on legal documents
RoBERTa-Legal	Large-level BPE tokenizer	512	32	2e-5	AdamW	Legal corpus pretraining
T5 (Raffel et al., 2020)	SentencePiece tokenizer	512	16	1e-5	AdaFactor	Contract extraction focus
SpanBERT-Legal (Bakker et al., 2025)	WordPiece tokenizer	512	32	2e-5	AdamW	Span-level representation
ContractNLI (Koreeda and Manning, 2021)	WordPiece tokenizer	512	32	2e-5	AdamW	Contract-focused legal adaptation
LegalNER.pt (Avram et al., 2021)	Portuguese legal terminology	512	16	1e-5	AdamW	Portuguese legal documents

By comparing LegNER against this spectrum of baselines, we isolate the contributions of legal-domain pretraining, span-level supervision, and vocabulary adaptation, ensuring that the reported improvements are both quantitative and interpretable.

### 5.4 Model fine-tuning

Fine-tuning adapts the pretrained backbone to the supervised task of legal NER. We adopt the standard BIO tagging scheme, where tokens are labeled as Beginning (B), Inside (I), or Outside (O) of entity spans. For example, “Judge Alice Thompson” is tagged as B-PERSON, I-PERSON, I-PERSON.

Tokenization is handled by the native tokenizer of each transformer: WordPiece for BERT variants and byte-pair encoding (BPE) for RoBERTa. Since legal entities often consist of multiword phrases (e.g., “Court of Justice of the European Union”), alignment strategies were applied to ensure accurate label propagation across subtokens. Specifically, the first subtoken inherits the BIO label, while subsequent subtokens are assigned continuation markers. This prevents boundary mismatches and reduces false negatives caused by fragmented legal terms.

The LegNER model builds on BERT-base with 12 transformer encoder layers, each with 768 hidden dimensions and 12 self-attention heads. Its vocabulary was initialized with 30,522 tokens and extended by 1,245 legal-specific terms extracted from EUR-Lex and curated dictionaries. This extension addresses vocabulary mismatch between general-purpose embeddings and domain-specific terminology, a known limitation of out-of-domain models in legal NLP.

To accommodate the long-form nature of legal documents, the maximum input sequence length was set to 512 tokens. This balances coverage of multi-paragraph judgments with memory efficiency. Hyperparameters were selected through empirical validation and guided by prior benchmarks in domain-adapted NER:

- Learning rate: 3*e*−5 with linear decay
- Batch size: 16
- Epochs: 8
- Optimizer: AdamW with weight decay of 0.01
- Maximum sequence length: 512
- Training hardware: NVIDIA A100 GPU (Google Colab Pro)

Early stopping was applied based on validation F1-score to mitigate overfitting and ensure stable generalization across entity classes.

### 5.5 Evaluation metrics

Model evaluation combined standard NER metrics with anonymization-oriented measures tailored to legal text processing. This dual perspective ensures that performance is assessed not only in terms of entity recognition accuracy but also in terms of suitability for GDPR-compliant anonymization workflows.

**Standard NER metrics**. Entity-level Precision, Recall, and F1-score were computed using strict span matching, requiring both entity boundaries and types to be correct:

- **Precision:**

$$\begin{array}{l} P=\frac{TP}{TP+FP} \end{array}$$ (1)

- **Recall:**

$$\begin{array}{l} R=\frac{TP}{TP+FN} \end{array}$$ (2)

- **F1-Score:**

$$\begin{array}{l} F1=2\cdot \frac{Precision\cdot Recall}{Precision+Recall} \end{array}$$ (3)

where TP = true positives, FP = false positives, and FN = false negatives. Strict boundary matching was applied to avoid partial credit for incomplete or misaligned entity spans.

**Anonymization-oriented metrics**. In addition to recognition accuracy, we introduced evaluation criteria specific to anonymization:

- **Effectiveness:** Measures the completeness of sensitive entity coverage in the anonymized text by comparing predictions against gold-standard annotations.
- **Consistency:** Assesses whether repeated mentions of the same entity are anonymized uniformly (e.g., “John Smith” → PERSON_1 throughout).
- **Readability:** Rates the semantic coherence and legal usability of anonymized documents, based on expert review with Likert-scale scores.
- **Consistency rate:** Quantifies uniformity of replacements across repeated mentions:

$$\begin{array}{l} \text{Consistency Rate}=\frac{\text{Consistent replacements}}{\text{Total replacements}} \end{array}$$ (4)

These domain-specific metrics capture properties that are critical for practical deployment. For example, high F1 alone does not guarantee reliable anonymization if the same entity is replaced inconsistently. By combining recognition accuracy with functional anonymization criteria, evaluation reflects both technical correctness and compliance readiness.

## 6 Experimental evaluation

This section presents a comprehensive assessment of LegNER across multiple dimensions of performance and usability. The evaluation framework is designed to capture not only quantitative recognition accuracy but also the models effectiveness in real-world legal anonymization scenarios. Specifically, we examine the experimental setup, compare LegNER against state-of-the-art legal NER baselines, analyze training dynamics and convergence behavior, assess entity-wise performance across six categories, and conduct a qualitative review of anonymized outputs. The section concludes with a discussion synthesizing the empirical findings and their implications for practical deployment in GDPR-compliant legal workflows.

### 6.1 Evaluation setup

Evaluation was conducted on a held-out test set comprising 232 court case documents, representing 15% of the full corpus of 1,542 cases. The dataset split followed a stratified sampling strategy, with 70% (1,079 documents) allocated to training, 15% (231 documents) to validation, and 15% (232 documents) to testing. Stratification ensured proportional representation of all six entity categories (PERSON, ORGANIZATION, LOCATION, DATE, LAW, CASE_REFERENCE) across subsets, reducing the risk of overfitting to frequent classes.

The test partition was used exclusively for final evaluation, with no overlap or leakage from training or validation stages. Model predictions were compared against gold-standard annotations using both standard NER metrics (precision, recall, and F1-score) and anonymization-oriented measures (effectiveness, consistency, readability, and consistency rate).

This setup provides a rigorous and unbiased assessment of LegNER under realistic conditions, approximating its deployment in anonymization of judicial decisions, compliance monitoring, and large-scale legal text analytics.

### 6.2 Comparative performance analysis

To benchmark LegNER, we compared it against state-of-the-art legal NER models under identical training and evaluation conditions. All baselines were fine-tuned on the same training split and evaluated on the same test set of 232 court case documents, ensuring a fair comparison. The baselines included **Legal-BERT**, **RoBERTa-Legal**, **SpanBERT-Legal**, **ContractNLI** (Koreeda and Manning, 2021), and **LegalNER.pt**, each representing different combinations of domain pretraining, tokenization strategies, and encoder architectures.

Table 3 reports results across six dimensions: accuracy, precision, recall, F1-score, inference speed (documents per second), and parameter size. Together these metrics capture both predictive performance and computational efficiency.

**Table 3:** Comparative evaluation of LegNER and baseline legal NER models.

**Model**	**Accuracy**	**Precision**	**Recall**	**F1 Score**	**Speed (docs/sec)**	**Parameters**
LegNER (Ours)	99.9%	99.5%	99.3%	99.4%	12.3	110M
Legal-BERT	97.8%	94.2%	93.1%	93.6%	10.1	110M
RoBERTa-legal	97.5%	93.8%	92.4%	93.1%	9.8	125M
ContractNLI	96.2%	91.1%	90.5%	90.8%	8.6	110M
SpanBERT-legal	96.9%	92.5%	91.3%	91.9%	8.9	125M
LegalNER.pt	95.4%	89.8%	88.9%	89.3%	11.4	110M

LegNER achieved the best results across all metrics. Its F1-score of 99.4% surpassed the next-best baseline, Legal-BERT, by almost 6 percentage points. The model also maintained a balanced precision (99.5%) and recall (99.3%), confirming robustness in minimizing both false positives and false negatives.

In terms of efficiency, LegNER processed 12.3 documents per second–faster than all baselines, including smaller models such as LegalNER.pt. This indicates that the performance gains were achieved without sacrificing inference speed or inflating model size, as LegNER maintains a comparable parameter count (110M) to most baselines.

The sharp contrast with LegalNER.pt is particularly notable. Despite having similar parameter size, LegalNER.pt underperformed by nearly 10 F1 points, highlighting the importance of high-quality, jurisdiction-specific training data and span-level annotation strategies. Other strong baselines such as SpanBERT-Legal and ContractNLI (Koreeda and Manning, 2021) also lagged behind, reinforcing that LegNER achieves a superior balance of accuracy and scalability.

Overall, these results establish LegNER as a new benchmark for legal NER, combining state-of-the-art accuracy with computational efficiency suitable for large-scale deployment.

### 6.3 Training dynamics and convergence behavior

The LegNER model demonstrated rapid convergence and stable learning behavior. From the first epoch onward, performance metrics improved consistently, indicating that the model effectively captured domain-specific linguistic patterns.

The progression of LegNERs performance during training was monitored across eight epochs, with evaluation metrics including accuracy, precision, recall, F1-score, and loss. This allows us to assess both convergence speed and training stability. The results are summarized in Table 4.

**Table 4:** Progression of evaluation metrics across eight training epochs for the LegNER model.

**Epoch**	**Accuracy**	**Precision**	**Recall**	**F1 Score**	**Loss**
1	99.3%	85.2%	99.5%	91.8%	0.022
2	99.9%	98.5%	92.3%	95.3%	0.001
3	99.9%	99.5%	99.3%	99.4%	0.0005
4	99.9%	99.5%	99.3%	99.4%	0.0005
5	99.8%	99.5%	99.3%	99.4%	0.0002
6	99.4%	99.5%	99.3%	99.4%	0.0005
7	99.9%	99.5%	99.3%	99.4%	0.0009
8	99.9%	99.6%	99.7%	99.4%	0.0006

As shown in Table 4, LegNER achieved rapid convergence within the first three epochs. Precision started relatively low at 85.2% in epoch 1 but improved sharply to 98.5% by epoch 2 and stabilized at 99.5% from epoch 3 onward. Recall displayed the opposite trajectory: it began at 99.5% in epoch 1, dropped to 92.3% in epoch 2 as the model adjusted its decision boundaries, and quickly recovered to 99.3% by epoch 3. This early trade-off between precision and recall reflects the models transition from over-predicting entities to refining boundaries with greater accuracy. The F1-score mirrored these dynamics, rising from 91.8% in epoch 1 to 95.3% in epoch 2, and reaching 99.4% from epoch 3 onward, where it remained stable through the remainder of training.

Loss values confirm this convergence pattern. The model began with a loss of 0.022 in epoch 1, dropped by an order of magnitude to 0.001 in epoch 2, and remained below 0.001 from epoch 3 forward. Importantly, the minimal fluctuations in loss across later epochs (0.0002–0.0009) indicate that the model achieved a stable equilibrium between learning and generalization. Unlike models that show oscillations or late-epoch divergence, LegNER maintained consistent optimization without overfitting.

Taken together, these results demonstrate that LegNER converges quickly, reaching near-optimal performance by the third epoch. Its ability to balance precision and recall, stabilize F1-score, and sustain low loss values across subsequent epochs confirms both learning efficiency and robustness. This behavior is particularly advantageous in legal NLP, where reliable convergence reduces computational costs while ensuring consistent entity recognition performance across complex legal documents.

### 6.4 Entity recognition performance

Beyond aggregate metrics, it is important to examine LegNERs ability to generalize across different types of legal entities. Entity-level analysis highlights strengths and weaknesses in recognizing diverse categories, ranging from personal identifiers to statutory references. Table 5 reports precision, recall, and F1-scores for all six annotated entity types.

**Table 5:** Entity-wise performance metrics on the test set.

**Entity Type**	**Precision**	**Recall**	**F1 Score**
PERSON	98.7%	99.1%	98.9%
ORGANIZATION	97.5%	96.8%	97.1%
LOCATION	98.3%	97.9%	98.1%
DATE	99.4%	99.6%	99.5%
LAW	97.8%	98.3%	98.0%
CASE_REFERENCE	98.9%	99.2%	99.0%

As shown in Table 5, LegNER achieved consistently strong results across all six entity types. The best performance was observed for DATE entities, with an F1-score of 99.5%. This reflects the models ability to accurately capture temporal expressions, which often appear in standardized formats (e.g., “15 March 2023”), making them less ambiguous for classification.

Similarly, CASE_REFERENCE and PERSON entities were recognized with near-perfect accuracy, achieving F1-scores of 99.0% and 98.9%, respectively. Reliable recognition of case identifiers is essential for citation tracking, while accurate detection of personal names is critical for anonymization workflows. The high recall values in these categories confirm the models ability to capture the majority of relevant mentions with minimal omissions.

**Example of entity anonymization.**

Original Text: On March 15, 2022, the plaintiff John Smith filed a complaint against Acme Corporation in the District Court of Brussels, represented by attorney Sarah Johnson from Legal Partners LLP. Anonymized Text: On [DATE-1], the plaintiff [PERSON-1] filed a complaint against [ORGANIZATION-1] in the District Court of [LOCATION-1], represented by attorney [PERSON-2] from [ORGANIZATION-2].

Slightly lower–but still strong–performance was recorded for ORGANIZATION (97.1% F1) and LAW (98.0% F1). These categories are inherently more challenging due to longer spans and greater lexical variation. Organizational entities often appear in compound forms (e.g., “European Commission Directorate-General for Competition”), which can lead to boundary errors. Similarly, legal statutes are referenced with varied formatting conventions, requiring the model to generalize across multiple syntactic patterns.

LOCATION entities achieved an F1 of 98.1%, confirming robustness in recognizing geographic references. Although performance was slightly below that of DATE and CASE_REFERENCE, the results remain sufficiently high to support reliable anonymization and semantic indexing in legal texts.

Overall, the entity-level analysis confirms that LegNER performs consistently well across all categories, including those with complex or variable surface forms. The combination of high precision and recall across diverse entities demonstrates its suitability for fine-grained legal NLP tasks such as anonymization, citation graph construction, and compliance monitoring.

### 6.5 Qualitative assessment

While quantitative evaluation provides evidence of predictive performance, qualitative analysis highlights how LegNER behaves in real-world anonymization scenarios. This perspective is essential for assessing not only accuracy but also the consistency, coherence, and readability of anonymized legal texts. An illustrative example is shown in the anonymization output of following Listing.

As shown in Listing, LegNER successfully detected and anonymized multiple sensitive entities, including personal names, organizational identifiers, geographic references, and dates. Entity replacements were applied systematically and consistently, preserving the original sentence structure and ensuring that the anonymized text remained legally coherent and intelligible.

A manual review of a subset of 12 anonymized case documents confirmed this behavior. LegNER achieved a consistency rate of 98.7%, meaning that repeated mentions of the same entity were anonymized uniformly throughout the document (e.g., “John Smith” → PERSON_1). This property is critical in legal workflows, as inconsistent replacements can undermine privacy protection and create interpretability issues in judicial texts.

Readability was also preserved. Legal experts rated the anonymized documents on a five-point Likert scale, where 1 indicated incoherence and 5 indicated full coherence. The average score was 4.8, showing that despite entity masking, the documents retained their semantic flow and remained suitable for research, compliance auditing, or public access under GDPR regulations.

Taken together, these findings demonstrate that LegNER not only performs well on standard NER metrics but also meets practical requirements for anonymization: high consistency in entity replacement, protection of sensitive information, and preservation of legal text readability. This makes the model directly applicable to GDPR-compliant redaction pipelines and broader legal NLP applications requiring privacy-aware document processing (Poplavska et al., 2020).

## 7 Discussion

The experimental evaluation highlights several important aspects of LegNERs effectiveness, ranging from learning dynamics to real-world applicability. This subsection synthesizes the findings to underline the models contributions and the implications for legal NLP and anonymization workflows.

First, the training dynamics demonstrated that LegNER converges rapidly, reaching near-optimal performance by the third epoch. The stability of precision, recall, and F1-scores across subsequent epochs indicates that the model effectively learned domain-specific entity structures without overfitting. This efficiency reduces training costs and confirms that the span-level annotation scheme and legal-domain vocabulary extensions provided high-quality input representations.

Second, the final evaluation metrics confirm that LegNER achieves state-of-the-art performance. With an accuracy of 99.9%, precision of 99.5%, recall of 99.3%, and F1-score of 99.4%, the model outperformed all baselines, including Legal-BERT, RoBERTa Legal NER, and SpanBERT. Importantly, the balance between precision and recall shows that LegNER minimizes both false positives and false negatives–a property that is especially valuable in anonymization, where both missed detections and spurious extractions pose risks for compliance.

Third, entity-level analysis provided evidence of LegNERs robustness across categories. Particularly strong results were observed for DATE, CASE_REFERENCE, and PERSON, which are essential for anonymization and legal citation tracking. Although categories such as ORGANIZATION and LAW showed slightly lower performance, they still achieved F1-scores above 97%, demonstrating the models capacity to generalize across complex entity types with varied lexical and syntactic patterns.

Fourth, the qualitative assessment confirmed that LegNER produces anonymized outputs that are both consistent and readable. Based on a 12-document subset, the model achieved a 98.7% consistency rate in replacing repeated mentions of the same entity, while expert evaluation yielded a 4.8/5 readability score. These results show that LegNER not only protects sensitive data but also preserves the interpretability of legal texts, supporting use in GDPR-compliant redaction pipelines and public access initiatives.

Finally, the combination of span-level annotations, transfer learning strategies, and feedback-driven optimization explains the observed performance gains. By integrating carefully curated datasets with domain-specific transformer adaptation, LegNER sets a new benchmark for legal NER and anonymization. Its design demonstrates how methodological choices–such as stratified sampling, vocabulary extensions, and BIO tagging–directly translate into gains in both technical performance and real-world usability.

In summary, the discussion of results shows that LegNER balances technical excellence with practical applicability. Its rapid convergence, high accuracy, consistent anonymization, and strong generalization across entity types establish it as a robust solution for legal NLP. These characteristics make the model well-suited for deployment in compliance-heavy environments, where both precision and trustworthiness are paramount.

## 8 Conclusions and future work

This study introduced **LegNER**, a transformer-based Named Entity Recognition (NER) system tailored for legal document processing and anonymization. By combining domain-specific fine-tuning, structured span-level annotations, and a rigorous evaluation protocol, LegNER achieved state-of-the-art performance in both predictive accuracy and practical anonymization. Across multiple metrics—including precision (99.5%), recall (99.3%), and F1-score (99.4%)—the model consistently outperformed strong baselines, confirming its robustness and generalization capacity. Qualitative assessments further demonstrated that LegNER produces anonymized outputs with high consistency and readability, making it suitable for GDPR-compliant pipelines and other high-stakes legal applications.

Our evaluation nevertheless highlights specific limitations that shape the next steps of this research. Although LegNER consistently achieved high precision and recall, a small number of failure cases remain, especially in very long, syntactically complex sentences. These edge cases confirm earlier psycholinguistic observations that sentence structure, more than specialized vocabulary, drives much of legal text complexity (Mart́ınez et al., 2022). Moreover, while the model generalized well within our multilingual EU dataset, further adaptation—through fine-tuning on jurisdiction-specific corpora and low-resource languages—is needed to ensure broader applicability across diverse legal systems (Krasadakis et al., 2024). By grounding future directions in these observed limitations, we ensure that subsequent improvements are directly linked to the empirical findings of this study.

Building on these observations, we outline several research avenues that naturally extend the present work. First, improving adaptability through methods such as few-shot learning or continual learning could reduce reliance on large annotated corpora and facilitate deployment in dynamic regulatory environments (Zhong et al., 2020). Second, given the globalized nature of legal practice, multilingual and cross-lingual NER systems represent a critical frontier. Resources such as the Leeds Arabic-English constitutional corpora (El-Farahaty et al., 2023) and ongoing efforts in multilingual legal corpora (Benaouda, 2023; Polo et al., 2021; Tena, 2020; Wu et al., 2023) provide valuable foundations, but challenges remain in harmonizing annotation standards and legal terminology across languages. Effective solutions in this area would support comparative law, cross-border litigation, and international compliance. Ensuring the accuracy and reliability of translations also remains crucial, especially in multilingual legal systems where inconsistency can undermine legal validity (Kupriyanova et al., 2023).

Equally important is the ethical dimension. Future NER systems must integrate mechanisms for transparency, bias mitigation, and human-in-the-loop oversight to ensure accountability and fairness in legal decision support (Wachter et al., 2017). Protecting privacy and preventing unintended information leakage are paramount, particularly in anonymization pipelines that process sensitive judicial data.

Finally, the integration of NER with other NLP technologies—such as legal question answering, argument mining, and document summarization—offers a pathway toward more comprehensive AI-assisted legal systems. Such combined capabilities would allow practitioners not only to identify entities but also to extract facts, summarize rulings, and answer domain-specific queries in context-aware ways. Surveys on legal judgment prediction highlight the growing dependence on structured representations as inputs for downstream tasks (Cui et al., 2023), underscoring the foundational role of models like LegNER. Incorporating user feedback and adapting to evolving corpora in real time could further enhance the long-term usability of legal NER systems.

In conclusion, LegNER advances the state of the art in legal NER by demonstrating that domain-adapted transformers can deliver both technical excellence and real-world applicability. Moving forward, collaboration between technologists and legal practitioners will be essential to align model design with professional standards and ethical imperatives, ensuring that AI supports transparency, fairness, and accountability in legal systems worldwide.

## Data Availability

The original contributions presented in the study are included in the article/supplementary material, further inquiries can be directed to the corresponding author.

## Footnotes

1 https://eur-lex.europa.eu.

2 https://huggingface.co/nlpaueb/legal-bert-base-uncased.

3 https://huggingface.co/Saibo-creator/legal-roberta-base.

4 https://huggingface.co/SpanBERT/spanbert-base-cased.

5 https://huggingface.co/tau/bart-base-sled-contractnli.

6 https://huggingface.co/dominguesm/legal-bert-ner-base-cased-ptbr.

## References

[B1] Alcántara Francia O. A. Nunez-del Prado M. Alatrista-Salas H. (2022). Survey of text mining techniques applied to judicial decisions prediction. Appl. Sci. 12:10200. doi: 10.3390/app122010200

[B2] Al-Jarf R. (2023). Problems of identifying lexical and syntactic features of legal documents by undergraduate efl students. J. Pragmat. Discourse Analy. 2, 16–24. doi: 10.32996/jpda.2023.2.1.3

[B3] Al-Qurishi M. AlQaseemi S. Soussi R. (2022). Aralegal-bert: A pretrained language model for arabic legal text. arXiv [preprint] arXiv:2210.08284. doi: 10.18653/v1/2022.nllp-1.31

[B4] Althammer S. Hofstätter S. Hanbury A. (2021). “Cross-domain retrieval in the legal and patent domains: A reproducibility study,” in 43rd European Conference on Advances in Information Retrieval (ECIR), volume 12657 of Lecture Notes in Computer Science (Cham: Springer), 3–17.

[B5] Alves A. Miranda P. Mello R. Nascimento A. (2023). “Automatic simplification of legal texts in portuguese using machine learning,” in Legal Knowledge and Information Systems (Amsterdam: IOS Press), 281–286.

[B6] Amato F. Fonisto M. Giacalone M. Sansone C. (2023). An intelligent conversational agent for the legal domain. Information 14:307. doi: 10.3390/info14060307

[B7] Andrade L. Becker K. (2023). “Bb25hlegalsum: Leveraging BM25 and bert-based clustering for the summarization of legal documents,” in 14th International Conference on Recent Advances in Natural Language Processing (RANLP) (Varna: INCOMA Ltd.), 255–263.

[B8] Avram A. Pais V. F. Tufis D. I. (2021). “Pyeurovoc: A tool for multilingual legal document classification with eurovoc descriptors,” in International Conference on Recent Advances in Natural Language Processing (RANLP) (Varna: INCOMA Ltd.), 92–101.

[B9] Bakker R. M. Schoevers A. J. van Drie R. A. N. Schraagen M. P. de Boer M. H. T. (2025). Semantic role extraction in law texts: a comparative analysis of language models for legal information extraction. Artif. Intellig. Law 2025, 1–35. doi: 10.1007/s10506-025-09437-x

[B10] Beebeejaun A. (2023). Plain language in mauritius: an empirical study of legal communication in mauritius. Int. J. Law Managem. 65, 172–188. doi: 10.1108/IJLMA-08-2022-0194

[B11] Benaouda A. (2023). Legal translation in algeria: the justice scales to the test of equivalence. ALTRALANG J. 5, 359–374. doi: 10.52919/altralang.v5i3.374

[B12] Bhattacharya P. Ghosh K. Ghosh S. Pal S. Ghosh S. Ghosh S. (2019). “Overview of the fire 2019 aila track: Artificial intelligence for legal assistance,” in Proceedings of the 11th Forum for Information Retrieval Evaluation (FIRE 2019) (New York, NY: ACM), 11–14.

[B13] Chalkidis I. Androutsopoulos I. Michos A. (2017). “Extracting contract elements,” in 16th International Conference on Artificial Intelligence and Law (ICAIL), 19–28.

[B14] Chalkidis I. Fergadiotis M. Malakasiotis P. Aletras N. Androutsopoulos I. (2020). “Legal-BERT: the muppets straight out of law school,” in Findings of the Association for Computational Linguistics: EMNLP (Stroudsburg, PA: Association for Computational Linguistics (ACL)), 2898–2904.

[B15] Chang L.-S. Malmasi S. Hosomura N. Zhang H. Brown C. J. Lei V. J. . (2021). Patient provider discussions of bariatric surgery and subsequent weight changes and receipt of bariatric surgery. Obesity 29, 1338–1346. doi: 10.1002/oby.23183 34111329

[B16] Cui J. Shen X. Wen S. (2023). A survey on legal judgment prediction: Datasets, metrics, models and challenges. IEEE Access 11, 102050–102071. doi: 10.1109/ACCESS.2023.3317083

[B17] Demertzis K. Rantos K. Magafas L. Skianis C. Iliadis L. (2023). A secure and privacy-preserving blockchain-based xai-justice system. Information 14:477. doi: 10.3390/info14090477

[B18] Dorfleitner G. Hornuf L. Kreppmeier J. (2023). Promise not fulfilled: Fintech, data privacy, and the gdpr. Electronic Markets 33:33. doi: 10.1007/s12525-023-00622-x

[B19] Dritsas E. Vonitsanos G. Livieris I. E. Kanavos A. Ilias A. Makris C. . (2019). “Pre-processing framework for twitter sentiment classification,” in International Conference on Artificial Intelligence Applications and Innovations (AIAI), volume 560 of IFIP Advances in Information and Communication Technology (Cham: Springer), 138–149.

[B20] Egger C. Caselli T. Tziafas G. de Saint Phalle E. de Vries W. (2024). Extracting and classifying exceptional covid-19 measures from multilingual legal texts: the merits and limitations of automated approaches. Regulat. Govern. 18, 704–723. doi: 10.1111/rego.12557

[B21] Eiermann M. (2024). The process of legal institutionalization: how privacy jurisprudence turned towards the us constitution and the american state. Law & Soc. Inquiry 49, 537–568. doi: 10.1017/lsi.2022.66

[B22] El-Farahaty H. Khallaf N. Alonayzan A. (2023). Building the leeds monolingual and parallel legal corpora of arabic and english countries constitutions: methods, challenges and solutions. Corpus Pragmatics 7, 103–119. doi: 10.1007/s41701-023-00138-x

[B23] Fan A. Wang S. Wang Y. (2024). Legal document similarity matching based on ensemble learning. IEEE Access 12, 33910–33922. doi: 10.1109/ACCESS.2024.3371262

[B24] Ferraro G. Lam H. Tosatto S. C. Olivieri F. Islam M. B. van Beest N. . (2019). “Automatic extraction of legal norms: evaluation of natural language processing tools,” in New Frontiers in Artificial Intelligence - *JSAI-isAI International Workshops, volume 12331 of Lecture Notes in Computer Science* (Cham: Springer), 64-81.

[B25] Garg M. Gajjar P. Shah P. Shukla M. Acharya B. Gerogiannis V. C. . (2023). Comparative analysis of deep learning architectures and vision transformers for musical key estimation. Information 14:527. doi: 10.3390/info14100527

[B26] Giampieri P. (2023). Is machine translation reliable in the legal field? A corpus-based critical comparative analysis for teaching esp at tertiary level. ESP Today 11, 119–137. doi: 10.18485/esptoday.2023.11.1.6

[B27] Giarelis N. Mastrokostas C. Karacapilidis N. (2023). Abstractive vs. extractive summarization: An experimental review. Appl. Sci. 13:7620. doi: 10.3390/app13137620

[B28] Giordano M. Cocco S. . (2023). Hate speech, incitamento allodio, incitación al odio: Eu parallel corpora, legal discourse, metadiscourse and translation. Int. J. English Linguist. 13, 1–21. doi: 10.5539/ijel.v13n5p1

[B29] Hua W. Zhang Y. Chen Z. Li J. Weber M. (2023). “Mixed-domain language modeling for processing long legal documents,” in Natural Legal Language Processing Workshop (Toronto, ON: Association for Computational Linguistics (ACL)), 51–61.

[B30] Huang P. Zhao X. Hu M. Fang Y. Li X. Xiao W. (2022). “Extract-select: a span selection framework for nested named entity recognition with generative adversarial training,” in Findings of the Association for Computational Linguistics: ACL (Cedarville, Ohio: Association for Computational Linguistics), 85-96.

[B31] Kanavos A. Antonopoulos N. Karamitsos I. Mylonas P. (2023a). “A comparative analysis of tweet analysis algorithms using natural language processing and machine learning models,” in 18th International Workshop on Semantic and Social Media Adaptation and Personalization (SMAP) (Limassol: IEEE), 1–6.

[B32] Kanavos A. Karamitsos I. Mohasseb A. Gerogiannis V. C. (2023b). “Comparative study of machine learning algorithms and text vectorization methods for fake news detection,” in 14th International Conference on Information, Intelligence, Systems & *Applications (IISA)* (Volos: IEEE), 1–8.

[B33] Kapoor A. Dhawan M. Goel A. Arjun T. Bhatnagar A. Agrawal V. . (2022). Hldc: Hindi legal documents corpus. arXiv [preprint] arXiv:2204.00806. doi: 10.18653/v1/2022.findings-acl.278

[B34] Katz D. M. Bommarito M. J. Gao S. Arredondo P. (2024). Gpt-4 passes the bar exam. Philosop. Trans. Royal Soc. A 382:0254. doi: 10.1098/rsta.2023.0254 38403056 PMC10894685

[B35] Koreeda Y. Manning C. D. (2021). Contractnli: A dataset for document-level natural language inference for contracts. arXiv [preprint] arXiv:2110.01799. doi: 10.18653/v1/2021.findings-emnlp.164

[B36] Krasadakis P. Sakkopoulos E. Verykios V. S. (2024). A survey on challenges and advances in natural language processing with a focus on legal informatics and low-resource languages. Electronics 13:648. doi: 10.3390/electronics13030648

[B37] Kupriyanova A. Shkilev R. Litwinowa M. Vorotilina T. Tagibov K. A. Shichiyakh R. (2023). Legal regulation of the reliability and quality of translations of official documents and texts. J. Law Sustain. Dev. 11, e1330–e1330. doi: 10.55908/sdgs.v11i11.1330

[B38] Leitner E. Rehm G. Moreno-Schneider J. (2019). “Fine-grained named entity recognition in legal documents,” in International Conference on Semantic Systems (Cham: Springer), 272–287.

[B39] Letychevskyi O. Peschanenko V. Poltorackiy M. Y. (2022). Algebraic approach to the analysis of legal documents. Prob. Program. 3–4, 117–127. doi: 10.15407/pp2022.03-04.117

[B40] Li Q. Zhang Q. (2021). “Court opinion generation from case fact description with legal basis,” in AAAI Conference on Artificial Intelligence (Palo Alto, CA: AAAI Press), 14840–14848.

[B41] Lin C. Cheng P. (2024). Legal documents drafting with fine-tuned pre-trained large language model. arXiv [preprint] arXiv:2406.04202. doi: 10.5121/csit.2024.140819

[B42] Martínez E. Mollica F. Gibson E. (2022). Poor writing, not specialized concepts, drives processing difficulty in legal language. Cognition 224:105070. doi: 10.1016/j.cognition.2022.105070 35257980

[B43] Mohasseb A. Kanavos A. Amer E. (2025). Enhancing text classification through grammar-based feature engineering and learning models. Information 16:424. doi: 10.3390/info16060424

[B44] Moreno-Schneider J. Rehm G. Montiel-Ponsoda E. Rodriguez-Doncel V. Revenko A. Karampatakis S. . (2020). Orchestrating NLP Services for the Legal Domain. Marseille: European Language Resources Association.

[B45] Mouratidis D. Mathe E. Voutos Y. Stamou K. Kermanidis K. L. Mylonas P. . (2022). “Domain-specific term extraction: A case study on greek maritime legal texts,”? in *12th Hellenic Conference on Artificial Intelligence (SETN)* (New York: ACM), 10:1–10:6. doi: 10.1145/3549737.3549751

[B46] Nguyen H.-T. Nguyen M.-P. Vuong T.-H.-Y. Bui M.-Q. Nguyen M.-C. Dang T.-B. . (2022). Transformer-based approaches for legal text processing. Rev. Socionetw. Strat. 16, 135–155. doi: 10.1007/s12626-022-00102-2

[B47] Oussalah M. (2021). “AI explainability. a bridge between machine vision and natural language processing,” in Pattern Recognition. ICPR International Workshops and Challenges (Cham: Springer), 257–273.

[B48] Paul J. (2023). Traditional knowledge protection and digitization: a critical decolonial discourse analysis. Int. J. Semiot. Law 36, 2133–2156. doi: 10.1007/s11196-023-09989-8

[B49] Paul S. Mandal A. Goyal P. Ghosh S. (2022). Pre-training transformers on indian legal text. arXiv [preprint] arXiv:2209.06049. doi: 10.48550/arXiv.2209.06049

[B50] Polo F. M. Mendonça G. C. F. Parreira K. C. J. Gianvechio L. Cordeiro P. Ferreira J. B. . (2021). LegalNLP - natural language processing methods for the brazilian legal language. arXiv [preprint] arXiv:2110.15709. doi: 10.5753/eniac.2021.18301

[B51] Poplavska E. Norton T. B. Wilson S. Sadeh N. (2020). “From prescription to description: mapping the gdpr to a privacy policy corpus annotation scheme,” in Legal Knowledge and Information Systems (Amsterdam: IOS Press), 243–246.

[B52] Raffel C. Shazeer N. Roberts A. Lee K. Narang S. Matena M. . (2020). Exploring the limits of transfer learning with a unified text-to-text transformer. J. Mach. Learn. Res. 21, 1–67. doi: 10.48550/arXiv.1910.1068334305477

[B53] Rodríguez-Puente P. Hernández-Coalla D. (2023). The corpus of contemporary english legal decisions, 1950-2021 (coceld): A new tool for analysing recent changes in english legal discourse. ICAME J. 47, 109–117. doi: 10.2478/icame-2023-0006

[B54] Rossi A. Palmirani M. (2015). “From words to images through legal visualization,” in International Workshop on AI Approaches to the Complexity of Legal Systems (Cham: Springer), 72–85.

[B55] Roufas N. Mohasseb A. Karamitsos I. Kanavos A. (2025). “Analyzing public discourse and sentiment in climate change discussions using transformer-based models,” in International Conference on Artificial Intelligence Applications and Innovations (AIAI), volume 754 of IFIP Advances in Information and Communication Technology (Cham: Springer), 39–53.

[B56] Ruggeri F. Lagioia F. Lippi M. Torroni P. (2022). Detecting and explaining unfairness in consumer contracts through memory networks. Artif. Intellig. Law 30, 59–92. doi: 10.1007/s10506-021-09288-2

[B57] Sang E. F. De Meulder F. (2003). Introduction to the CoNLL-2003 Shared Task: Language-Independent Named Entity Recognition. Ithaca, NY.

[B58] Savelka J. Ashley K. D. (2023). The unreasonable effectiveness of large language models in zero-shot semantic annotation of legal texts. Front. Artif. Intellig. 6:1279794. doi: 10.3389/frai.2023.1279794 38045764 PMC10690809

[B59] Sharma A. Ghosh K. Bhattacharya P. Ghosh S. (2018). “Named entity recognition for indian legal documents,”? in *Proceedings of the LREC 2018 Workshop on Language Resources and Technologies for Indian Languages (LRT4IL)* (Paris: European Language Resources Association (ELRA)).

[B60] Sinyakin I. Samorodova E. Belyaeva I. (2020). Linguistic analysis of the peculiarities of the french-language legal task texts. XLinguae 13, 17–32. doi: 10.18355/XL.2020.13.02.02

[B61] Skianis K. Malliaros F. D. Tziortziotis N. Vazirgiannis M. (2020). “Boosting tricks for word movers distance,”? in *International Conference on Artificial Neural Networks* (Cham: Springer), 761–772.

[B62] Song D. Gao S. He B. Schilder F. (2022). On the effectiveness of pre-trained language models for legal natural language processing: an empirical study. IEEE Access 10, 75835–75858. doi: 10.1109/ACCESS.2022.3190408

[B63] Stow M. T. Ugwu C. Onyejegbu L. (2023). An improved model for legal case text document classification. Eur. J. Elect. Eng. Comp. Sci. 7, 58–64. doi: 10.24018/ejece.2023.7.2.509

[B64] Sugathadasa K. Ayesha B. de Silva N. Perera A. S. Jayawardana V. Lakmal D. . (2019). “Legal document retrieval using document vector embeddings and deep learning,” in Intelligent Computing: Proceedings of the 2018 Computing Conference (Cham: Springer), 160–175.

[B65] Tena F. G. (2020). Multilingual analysis of macrostructure in online lower court judgments of england and wales, Germany, France and Spain: a comparative summary and phraseology. Int. J. Linguist. Literat. Transl. 3, 129–139. doi: 10.32996/ijllt.2020.3.10.15

[B66] Tsarapatsanis D. Aletras N. (2021). On the ethical limits of natural language processing on legal text. arXiv [preprint] arXiv:2105.02751. doi: 10.18653/v1/2021.findings-acl.314

[B67] Tuggener D. von Däniken P. Peetz T. Cieliebak M. (2020). “LEDGAR: A large-scale multi-label corpus for text classification of legal provisions in contracts,” in 12th Language Resources and Evaluation Conference (LREC) (Luxembourg: European Language Resources Association), 1235–1241.

[B68] van der Veen M. Sidorova N. (2021). “Signal phrase extraction: A gateway to information retrieval improvement in law texts,” in Legal Knowledge and Information Systems (Amsterdam: IOS Press), 127–130.

[B69] Vonitsanos G. Kanavos A. Mylonas P. (2023). “Decoding gender on social networks: An in-depth analysis of language in online discussions using natural language processing and machine learning,” in IEEE International Conference on Big Data (Sorrento: IEEE), 4618–4625.

[B70] Wachter S. Mittelstadt B. Russell C. (2017). Counterfactual explanations without opening the black box: automated decisions and the GDPR. Harvard J. Law Technol. 31, 841–887. doi: 10.2139/ssrn.3063289

[B71] Wolf T. Debut L. Sanh V. Chaumond J. Delangue C. Moi A. . (2020). “Transformers: State-of-the-art natural language processing,” in Conference on Empirical Methods in Natural Language Processing: System Demonstrations (Stroudsburg, PA: Association for Computational Linguistics (ACL)), 38–45.

[B72] Wu Y. Zhou S. Liu Y. Lu W. Liu X. Zhang Y. . (2023). Precedent-enhanced legal judgment prediction with llm and domain-model collaboration. arXiv [preprint] arXiv:2310.09241. doi: 10.18653/v1/2023.emnlp-main.740

[B73] Xie Y. Li Z. Yin Y. Wei Z. Xu G. Luo Y. (2024). Advancing legal citation text c lassification a CONV1D-based approach for multi-class classification. J. Theory Pract. Eng. Sci. 4, 15–22. doi: 10.53469/jtpes.2024.04(02).03

[B74] Yang H. Hadjiantoni S. Long Y. Petraityte R. Lausen B. (2023). Automatic detection of industry sectors in legal articles using machine learning approaches. arXiv [preprint] arXiv:2303.05387.

[B75] Zhong H. Xiao C. Tu C. Zhang T. Liu Z. Sun M. (2020). “How does NLP benefit legal system: a summary of legal artificial intelligence,” in 58th Annual Meeting of the Association for Computational Linguistics (ACL) (Kerrville, TX: Association for Computational Linguistics), 5218–5230.

